# Explainable hybrid CNN-transformer with self-supervised learning for structural analysis of paranasal sinus CT

**DOI:** 10.3389/fncom.2026.1778260

**Published:** 2026-05-13

**Authors:** Najeeb Ullah, Shabbab Ali Algamdi, Tariq Sadad

**Affiliations:** 1 College of Computer and Systems Engineering, Abdullah Al Salem University, Khaldiya, Kuwait; 2 Department of Software Engineering, Prince Sattam bin Abdulaziz University, Saudi Arabia; 3 Department of Computer Science, University of Engineering and Technology, Mardan, Pakistan

**Keywords:** autoencoder residual learning, disease detection, explainable AI, medical image analysis, transformer-based segmentation

## Abstract

**Introduction:**

The process of precise structural evaluation for paranasal sinuses based on CT scan data establishes a foundation for medical professionals to assess human anatomical variations, supporting the diagnosis and treatment of ear, nose, and throat (ENT) conditions. Existing deep learning methods face difficulties in analyzing complex sinus structures due to limited annotated datasets and lack of clinical interpretability.

**Methods:**

This study presents an explainable hybrid CNN-Transformer framework incorporating a self-supervised 3D convolutional autoencoder to perform structural analysis of paranasal sinus CT volumes. The framework is evaluated on the multi-institutional CT-SCOPE dataset, which contains diverse scan data from different hospitals and CT scanner models. The proposed approach combines anatomical segmentation to generate precise boundaries with residual-based structural representation learning for anomaly detection without requiring pathology labels.

**Results:**

The hybrid segmentation model achieves high anatomical fidelity, producing Dice similarity coefficients above 0.83 across all four sinus regions, including maxillary, ethmoid, frontal, and sphenoid sinuses. The architecture integrates convolutional feature extraction with Transformer-based contextual modeling to capture both fine structural details and global anatomical context. The self-supervised autoencoder generates reconstruction residual maps that highlight structural deviations from standard osseous patterns. Grad-CAM-based interpretability analysis demonstrates that the model focuses on clinically relevant regions such as sinus walls and ethmoidal partitions.

**Discussion:**

The integration of accurate segmentation, residual-based structural representation learning, and explainable attention mechanisms provides a comprehensive framework for anatomical analysis of paranasal sinus CT images. This approach establishes a foundation for future pathology-aware clinical modeling and supports the development of reliable AI-assisted diagnostic systems.

## 1 Introduction

The maxillary, frontal, sphenoid, and ethmoid sinuses are air-filled cavities collectively known as the paranasal sinuses. These structures perform important physiological functions, including humidification of inhaled air, regulation of craniofacial temperature, and contribution to vocal resonance (Taxy, 1998). Structural and inflammatory changes in the paranasal sinuses are commonly observed in medical imaging, where mucosal thickening, cysts, and varying degrees of sinus opacification are frequently associated with acute and chronic sinusitis (Chang et al., 2022; Ikubor et al., 2021; Papadopoulou et al., 2021). Computed tomography (CT) is the primary imaging modality for sinus evaluation, as it provides detailed visualization of osseous anatomy and soft tissue structures, enabling accurate assessment of disease severity and progression (Noorian and Motaghi, 2012). Inter-patient anatomical variability combined with different tissue densities and incidental findings that resemble pathological conditions makes it difficult to interpret sinonasal CT scans (Hagtvedt et al., 2003). Manual or semi-automated segmentation techniques make up traditional sinus analysis methods because they need extensive expert knowledge and take a long time to perform (Pirner et al., 2009). Deep learning methods have become popular because they can solve the existing problems. Convolutional neural networks (CNNs) have demonstrated strong performance in sinus-related diagnostic tasks, including automated sinusitis detection using radiographs (Jeon et al., 2021; Kim et al., 2019). Researchers have investigated volumetric CT-based analysis and surgical planning techniques together with computer-assisted navigation methods to improve both sinus visualization and guidance during surgery (Lapeer et al., 2008; Lee et al., 2021; Lim et al., 2017; Linxweiler et al., 2020; Pallanch et al., 2013; Soler et al., 2015). Researchers have developed deep learning-based segmentation methods that use CNN architectures to automatically define sinus structures in CT scans (Iwamoto et al., 2019; Kuo and Liu, 2022; Kuo et al., 2022). The methods can successfully identify anatomical boundaries yet their performance as single-center small datasets from singular sites fails to produce satisfactory results because of its limited variation in testing environments. Recent studies have explored more advanced architectures, including hybrid CNN–Transformer models, which combine local feature extraction with global contextual reasoning. The hybrid architectures improve segmentation performance beyond conventional CNN-based methods according to Song et al. (2025) study results. Semantic segmentation methods that identify osseous sinus structures have undergone additional improvements to achieve better anatomical precision (Sun et al., 2025). The system has experienced progress, yet it still has multiple unresolved issues. Current research works assess segmentation performance as a separate process from clinical outcomes, which leads to incomplete connections between anatomical modeling and diagnostic interpretation. The restricted access to extensive annotated datasets from multiple institutions hinders successful model generalization and clinical applications.

To overcome data limitations, representation learning techniques have been explored, including reconstruction-based approaches using convolutional autoencoders (CAEs). These methods enable structural pattern learning through data reconstruction while the residual analysis process identifies data deviations which reveal anatomical structure differences (Song et al., 2025; Sun et al., 2025). The current techniques fail to support complex 3D volumetric medical data while their segmentation framework integration for anatomically guided analysis remains unfulfilled. The CT-SCOPE dataset introduction represents a significant progress point for solving these particular challenges (Sun et al., 2025). This multi-institutional dataset includes high-resolution CT scans and annotated segmentation masks of major paranasal sinus compartments, collected from multiple hospitals and scanner types. The diverse collection environment enables researchers to create and test their models across different conditions while developing more powerful generalizable models. The dataset’s existence enables researchers to repeat their studies while making their research methods open to public evaluation. The contributions of this paper are summarized as follows:

- A unified deep learning framework that integrates hybrid CNN–Transformer-based segmentation with self-supervised structural representation learning for accurate CT-based modeling of paranasal sinuses.
- A robust segmentation backbone that combines Swin-UNETR and CoTr architectures, leveraging both convolutional and Vision Transformer mechanisms to accurately delineate multiple anatomical sinus compartments in the CT-SCOPE dataset.
- A reconstruction-driven convolutional autoencoder (CAE) for self-supervised anomaly detection, which learns normal osseous sinus morphology and identifies structural variations through residual mapping without requiring pathology annotations.
- An explainability module based on Grad-CAM, generating anatomically consistent heatmaps that enhance model interpretability and support clinical understanding.

## 2 Literature review

The human body contains four pairs of paranasal sinuses, namely the maxillary, frontal, sphenoid, and ethmoid sinuses, which are air-filled cavities exhibiting significant anatomical variability. The differences between septal development and structural formation result in variations that make radiological interpretation difficult (Liu et al., 2022; Taxy, 1998). The different patterns of imaging density become visible through this variability, which creates obstacles for diagnosing acute and chronic rhinosinusitis along with other inflammatory conditions. The typical radiological findings show three main patterns, which include mucosal thickening and fluid accumulation and complete sinus opacification (Ikubor et al., 2021; Kim et al., 2022). Chronic sinus conditions induce tissue changes that complicate the differentiation between normal and abnormal anatomical structures. The clinical setting of endoscopic sinus surgery (ESS) requires accurate sinus anatomical identification because any structural mistakes involving the optic nerve or internal carotid artery can result in serious medical issues (Bhattacharya et al., 2022). Traditional methods depend on manual or semi-automated segmentation techniques, which require expert involvement to perform time-consuming work. Automated computational methods need to be developed because current methods face challenges due to unpredictable environmental conditions. The earlier deep learning techniques for paranasal sinus segmentation used convolutional neural networks (CNNs) to process 2D and 3D encoder–decoder architectures, including U-Net and its variants, which successfully outlined anatomical boundaries in CT scans (Iwamoto et al., 2019; Kuo and Liu, 2022; Kuo et al., 2022). The methods developed using small datasets from single institutions and specific sinus regions had limited applicability for different medical environments. More advanced architectures have been developed since their introduction. Song et al. (Song et al., 2025) conducted a thorough assessment of CNNs, vision transformers (ViTs), and hybrid models (e.g., Swin-UNETR), showing that CNN–Transformer hybrid systems successfully combine local feature extraction with long-range contextual modeling capabilities. The research shows three main issues with the studied systems, which include limited dataset variety and missing multi-center verification and restricted testing for difficult clinical situations that include severe opacification and anatomical differences. The transformer-based models use self-attention mechanisms to establish global context (Khan et al., 2022), but they need extensive computational capacity and complete large-scale annotated datasets for their operation. Deep learning studies have surveyed multiple paths for research, including 2D and 3D CNN methods for sinus disease classification (Bhattacharya et al., 2022; Jeon et al., 2021; Kim et al., 2019; Kim et al., 2022; Liu et al., 2022), while earlier work relied on manual segmentation methods (Pirner et al., 2009). The models showed good diagnostic skills, but their lack of specific anatomical segmentation made it hard for doctors to assess results and trust their clinical application. Model predictions can become affected by nearby body parts, which include the orbit and skull base and nasal cavity. Explainability techniques such as Grad-CAM and saliency maps do not ensure that the shown areas represent clinically significant anatomical or pathological features. Self-supervised learning (SSL) with its ability to create representations from medical images without requiring labeled data has become a suitable method for medical imaging. Contrastive learning and reconstruction-based approaches have demonstrated strong performance; however, their adaptation to 3D volumetric CT data remains challenging due to the need for preserving spatial consistency and capturing volumetric contextual information (Chen et al., 2020; Grill et al., 2020; He et al., 2022; Zhou et al., 2019). Anomaly detection methods use 3D convolutional autoencoders (CAEs) to find structural changes through the detection process of reconstruction errors (Song et al., 2025; Sun et al., 2025). The methods work for clinical decision-making, but they cannot be used in medical classification assessments. The CT-SCOPE dataset (Sun et al., 2025) launch provides a vital advance for semantic segmentation of paranasal sinus structures between different institutions. The dataset enables model testing under various conditions because it integrates different scanner types and acquisition methods and reconstruction processes. The dataset suffers from multiple drawbacks because it contains few complete annotated cases and lacks pathology-specific identification. The method cannot be used for disease diagnosis or predictive modeling because of its existing restrictions. Anomaly detection needs to be combined with segmentation and representation learning in integrated systems to make clinical analysis of 3D sinus CT data more effective. Table 1 provides an overview of existing paranasal sinus analysis methods that include segmentation and classification and detection and dataset contributions.

**TABLE 1:** Summary of representative studies on paranasal sinus analysis, categorized by task, methodology, and limitations.

References	Task	Method type	Modality	Key contribution	Limitation
Pirner et al. (2009)	Segmentation	Manual	CT	Manual delineation of sinus structures	Time-consuming, user-dependent
Jeon et al. (2021)	Classification	CNN (multi-view)	X-ray	Multi-view sinusitis diagnosis	No anatomical segmentation
Kim et al. (2019)	Classification	CNN	X-ray	Maxillary sinusitis detection	Limited generalization
Kuo and Liu (2022)	Segmentation	CNN	CT	Fully automatic sinus segmentation	Limited dataset diversity
Iwamoto et al. (2019)	Segmentation	CNN	CT	Automatic sinus segmentation	Single-center validation
Kuo et al. (2022)	Segmentation	Semi-supervised DL	CT	3D semantic segmentation	Requires partial annotations
Song et al. (2025)	Segmentation	CNN + transformer	CT	Benchmark of hybrid architectures	Limited clinical validation
Sun et al. (2025)	Segmentation	CNN	CT	Osseous structure segmentation	Focus on bone structures only
Liu et al. (2022)	Classification	Deep learning	CT	Tumor classification	No segmentation integration
Kim et al. (2022)	Detection	3D CNN	CT	Fungal sinus detection	Limited interpretability
Bhattacharya et al. (2022)	Classification	Contrastive learning (SSL)	CT	Anomaly classification	No structural localization

## 3 Materials and methods

The section presents the dataset for the study, which describes the process used to harmonize and pre-process multi-institutional CT volumes before the introduction of the unified deep learning framework. The ENT radiology practice uses a complete pipeline that includes three main functions: anatomical segmentation and self-supervised anomaly localization and segmentation-guided structural representation learning to support reliable performance across different imaging conditions. The framework concentrates on anatomical modeling and structural representation learning because CT-SCOPE lacks pathology labels for supervised disease classification.

### 3.1 Dataset

The proposed framework has been developed and evaluated with the CT-SCOPE dataset (Sun et al., 2025), the publicly available, multi-institutional CT repository specially created for semantic segmentation of paranasal sinus structures. The CT-SCOPE data-base establishes clear guidelines for creating anatomical models of the maxillary frontal ethmoidal and sphenoidal sinuses. The database contains 40 volumetric CT scans that were obtained from 6 tertiary hospitals using 4 different CT scanner models which includes Canon Aquilion GE LightSpeed VCT GE HiSpeed Dual and Siemens SOMATOM H70s. The scans were reconstructed at a 512 × 512 in-plane resolution between 0.47 and 1.0 mm slice thickness which includes the complete anatomical area of the paranasal sinuses. The varying scanner characteristics and reconstruction kernels and patient populations create a realistic radio-logical environment which makes CT-SCOPE suitable for developing algorithms that can be used in various clinical situations. Segmentation experts analyzed 13 of the total 40 subjects to create 696 pixel-level manual labels. The annotations define all major paranasal sinus compartments by using an exact one-pixel contour method which maintains anatomical precision. The system provides 1,031 pseudo-labels which were produced through an ensemble mechanism that uses deep learning technology. The pseudo-labels increase the training dataset size which enables the use of semi-supervised learning methods through two methods including the addition of unlabeled volumes and the enhancement of structural prior robustness.

### 3.2 Preprocessing pipeline

The entire CT-SCOPE volume underwent a standardized preprocessing workflow that accounted for consistency among subjects while aiming to enhance the input quality for segmentation and subsequent representation learning. The original dataset consists of DICOM series, which were converted into either PNG or NumPy array formats to facilitate fast loading during training. Invalid voxel intensities (e.g., NaN or ± ∞) were replaced with zero to guarantee numerical stability. Due to the small and varying proportion that the sinus cavities take up in the craniofacial region, resampling of each CT volume was done to an isotropic voxel spacing (1.0 × 1.0 × 1.0 mm^3^) and resized to 256 × 256 × 128 voxels for segmentation. An intensity normalization was performed with min–max scaling mapping Hounsfield unit values into the 0–1 range. By this standardization, scanner-induced intensity variability is reduced while being faster and more stable for optimization. Above the desired size, volumes were center-cropped to keep the sinus region intact, while smaller volumes were zero-padded symmetrically. Mask-guided cropping was used to remove structures such as the cranial vault and brain tissue that were irrelevant to the study. The background was suppressed by using an intensity threshold of −500 HU, thereby retaining osseous and soft-tissue regions pertinent to sinus anatomy. Although CT-SCOPE does not include soft-tissue diagnostic labels, care was exercised in applying spatial augmentations so that the underlying anatomy was not compromised. Non-geometric changes (gamma correction, intensity rescaling, etc.) were the only alterations undertaken to avert sinus morphology distortion or modification of pertinent structural boundaries for segmentation and subsequent feature extraction.

### 3.3 Proposed unified deep learning framework

**FIGURE 1:**
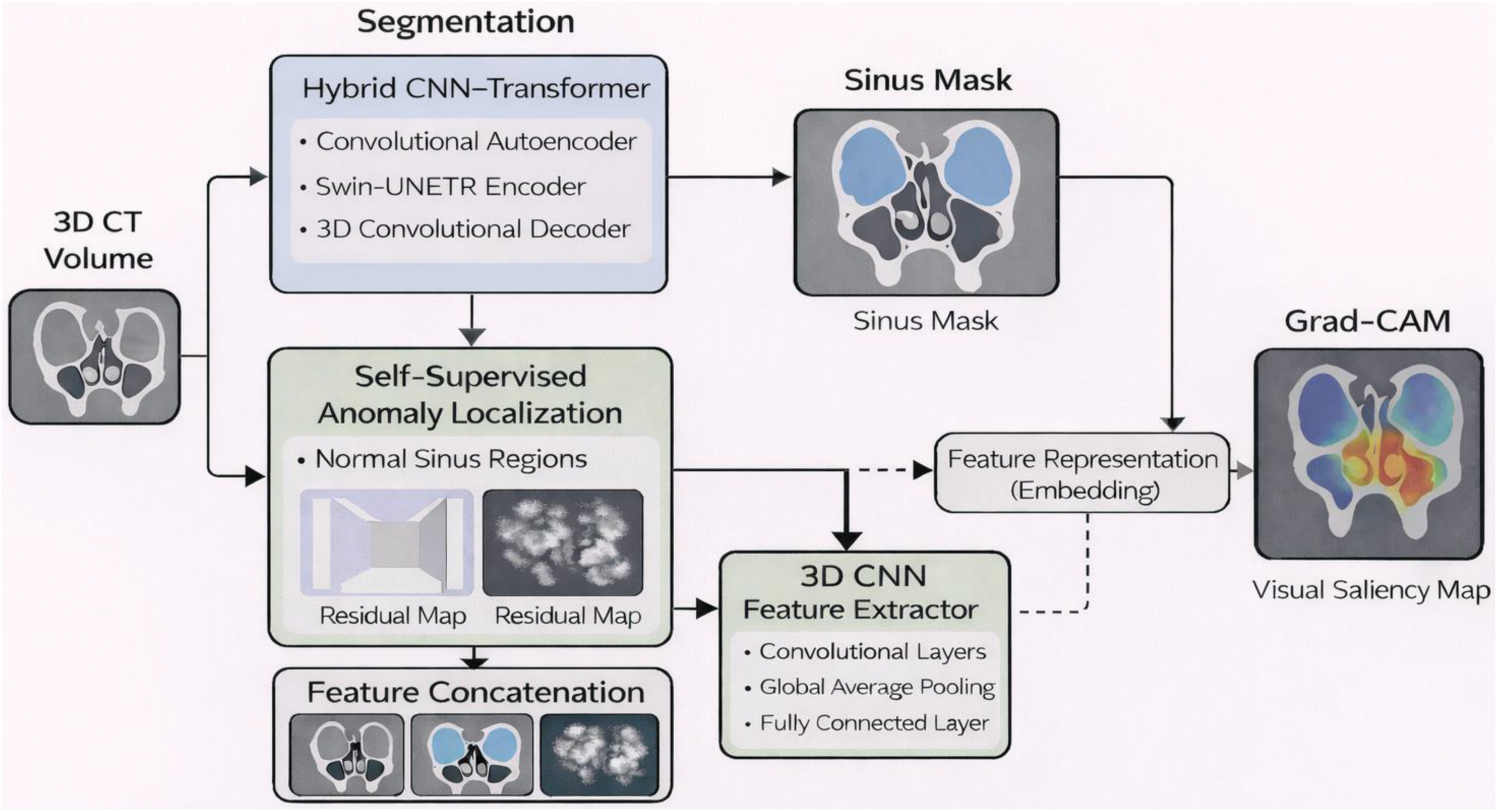
Overview of the proposed unified deep learning framework.

The proposed architecture consists of three main components: (i) a hybrid CNN-Transformer segmentation model that delineates paranasal sinus compartments; (ii) a self-supervised 3D convolutional autoencoder (CAE), which acquires the manifold of normal osseous sinus anatomy and recognizes deviations through reconstruction residuals; and (iii) a 3D convolutional network that extracts discriminative structural representations using segmentation-guided regions and CAE-derived residuals as shown in Figure 1. CT-SCOPE does not have pathological labels, but the combination of anatomical masks and voxel-wise residual retrieves value representations specific to common anatomical variance and odd structural patterns. This pipeline uses the first CT volume preprocessed from the segmentation backbone, producing specific sinus masks. This CAE also trains on anatomically consistent sinus regions to form a compact latent representation of healthy osseous morphology. Then during inference, differences between the input volume and its CAE reconstruction yield residual maps that highlight anatomical deviations or atypical variations. These maps together with the CT volume and segmentation mask are concatenated to form a multi-channel input to the 3D CNN feature extractor, which learns structural representations of sinus morphology rather than performing supervised disease classification. The final convolutional layer will then be subjected to Grad-CAM, which will generate interpretable saliency maps very likely on the basis of which sinus subregions contribute the most to the learned structural representations.

### 3.4 Hybrid CNN–transformer segmentation model

**FIGURE 2:**
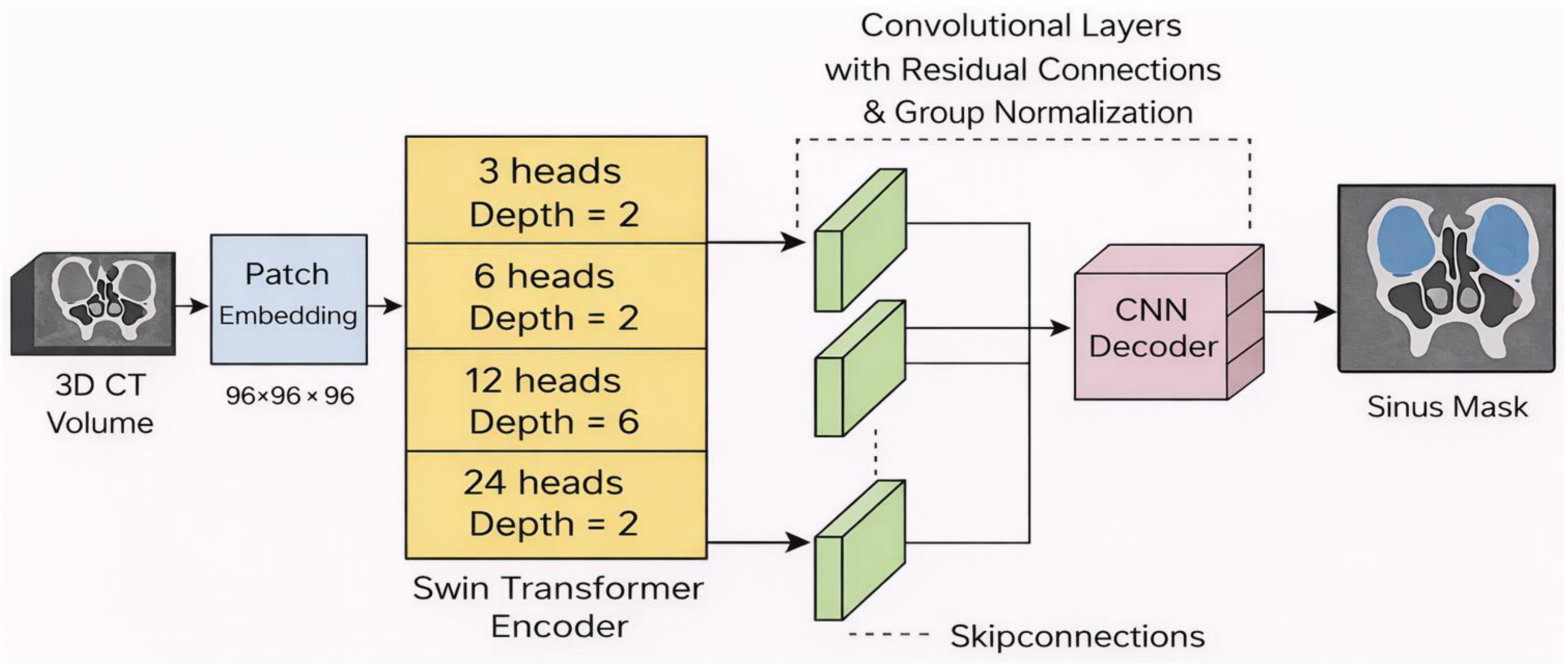
Segmentation model architecture.

The segmentation backbone is inspired by Swin-UNETR and CoTr, combining the strengths of window-based self-attention with convolutional texture extraction. The encoder utilizes hierarchical Swin Transformer blocks with shifted-window self-attention to capture both local and global dependencies. Patch embeddings of size 96 × 96 × 96 are passed through multi-head attention layers gradually increasing in head count (3, 6, 12, and 24) in a depth configuration of (2, 2, 6, 2). In the process, 3D convolutional layers with residual connections and group normalization are interleaved with Transformer blocks so that local boundary information can be enhanced. The decoder reconstructs multi-scale features using transposed convolutions and skip connections to ensure the fine anatomical details, mainly related to the thin bony walls of the ethmoid and maxillary sinuses, are retained as illustrated in Figure 2. Composite loss functions were used during training, combining Dice loss and cross-entropy, with equal weights. Optimization was performed using AdamW (with a learning rate of 1 × 10^−4^) for 200 epochs. Only intensity augmentations were applied during training to keep the sinus anatomy from suffering any geometric distortions.

We give more architectural and training specifications in order to be reproducible. The CNN-Transformer type of segmentation model is trained on a Swin Transformer block with a window size of 7 × 7 × 7 and hierarchical patch akinness of 96 × 96 × 96 voxels. The convolutional layers are alternated with transformer blocks, using kernels of 3 × 3 × 3, 32, 64, 128, and 256 filters at the consecutive steps, and group normalization is used after each convolution. This model is trained based on AdamW with an initial learning rate of 1 −10, cosine annealing learning rate adjustment, and a batch size of 2 during 200 epochs. The 3D CAE has 3 × 3 × 3 convolution, 32, 64, and 128 filters in the encoder and decoder, a latent vector dimension of 256, and voxel-wise L2 loss to do the reconstruction. The CAE is trained using Adam, an initial learning rate of 1 × 10 ^–3^, a batch size of 4, and a step-wise learning rate decrease after every 50 epochs. This information enables complete replication of both the segmentation and CAE training processes, retaining anatomy and the self-directed learning model of the model.

### 3.5 CAE-based self-supervised anomaly localization

A 3D convolutional autoencoder was trained on structurally normal regions solely extracted from CT-SCOPE sinus masks. Input volumes of size 64 × 64 × 64 were aligned and normalized before passing into the encoder. Encoder architecture composed of successive convolutional blocks with 32, 64, and 128 filters finally converged into a bottleneck latent vector of dimension 256. The decoder then reconstructs the input volume accurately through symmetric deconvolution layers. The CAE was trained using voxel-wise L2 loss to enforce the proper reconstruction of normal osseous anatomy. The absolute difference between the input and the reconstructed volume was used to create residual maps which were obtained during the inference process. The residual maps show areas of abnormal anatomical development which CT-SCOPE uses to identify pathology despite its lack of specific disease labels.

### 3.6 3 D CNN feature extraction network

The network is designed to learn morphology-aware structural embeddings rather than perform supervised classification, since CT-SCOPE does not provide pathology labels. The 3D CNN receives a three-channel input consisting of: (i) the preprocessed CT volume; (ii) the corresponding segmentation mask; and (iii) the CAE-derived residual map. This design encourages the network to focus on anatomically meaningful sinus regions and structural deviations. The system consists of three convolutional layers which have 32 filters and 64 filters and 128 filters, and these layers use max-pooling before proceeding to global average pooling. The 64-unit dense layers with 0.3 dropout rate create compact latent features which work for visualization and clustering and downstream tasks, even though CT-SCOPE lacks disease labels. The sinus morphology embeddings represent structural changes which medical researchers can use to build diagnostic models when clinical annotated datasets become accessible.

### 3.7 Integration of segmentation, representation learning, explainability, and evaluation metrics

The proposed framework depends on three essential elements which are segmentation and self-supervised representation learning and model interpretability. The hybrid CNN–Transformer backbone produces sinus segmentation masks which deliver medically accurate results by defining the boundaries of maxillary and ethmoid and frontal and sphenoid sinuses while omitting unneeded facial bone details. The following modules manage their operations inside spaces which doctors can use while background noise does not interfere with their work. The 3D convolutional autoencoder generates residual maps which display structural information by showing how bone structures in sinus anatomy differ from standard patterns. The irregular pneumatization patterns and asymmetric boundaries and other morphological variations create deviations which function as valuable informants for representation learning because these patterns exist without any specific pathology labels. The 3D CNN establishes its ability to differentiate structural elements through the combination of CT volume and segmentation mask and CAE-derived residual map. The CT-SCOPE dataset lacks diagnostic labels yet the representations found in this dataset possess the potential to assist with classification tasks when combined with clinically annotated datasets. Grad-CAM visualized the complete learning process of the 3D CNN through its final convolutional layer to show which regions most strongly contributed to developing learned representations. The resulting heatmaps consistently localized to clinically meaningful anatomical regions, including the maxillary sinus walls, ethmoidal air cells, frontal recess, and sphenoid sinus boundaries. The model uses anatomical features to boost its interpretation abilities because its focus on these features boosts determination about model results. The evaluation of segmentation performance depended on two key metrics which were the Dice Similarity Coefficient (DSC) and the 95th percentile Hausdorff distance (HD95). The learned representations from CT-SCOPE were assessed through visual assessment because the pathology labels were not present in the dataset. The proposed framework demonstrates both anatomical accuracy and structural stability through its two evaluation methods which comply with CT-SCOPE dataset restrictions.

## 4 Results

The proposed framework was tested using the CT-SCOPE dataset to assess its performance through five major criteria which included segmentation accuracy and qualitative anatomical fidelity and residual-based structural representation learning and Grad-CAM interpretability and testing against established baseline performance. The representation learning results for CT-SCOPE are presented through anatomical discriminability and feature localization which were achieved using fused input representations because the dataset lacks diagnostic labels.

### 4.1 Segmentation performance

A quantitative assessment of the proposed hybrid CNN–Transformer architecture for segmentation was conducted, demon-strating strong performance across all four major CT-SCOPE paranasal sinus compartments. The model achieved a mean Dice score of 0.84 and a mean Jaccard Index of 0.73, indicating accurate delineation of both coarse and fine anatomical structures. The maxillary sinuses produced the highest Dice score of 0.87 because of their larger size and better-defined cortical margins which create more distinct CT imaging results. The model achieved a Dice score of 0.84 for the ethmoid sinuses which have complex shapes that include multiple thin bony septations thus demonstrating its ability to handle difficult anatomical areas. The frontal and sphenoid sinuses demonstrated slightly reduced accuracy because they achieved Dice scores of 0.81 and 0.83, respectively. Table 2 shows how variations in frontal sinus pneumatization and slight boundary uncertainties in deeper sphenoid regions resulted in the observed differences. The system achieved balanced segmentation capabilities because all structures demonstrated precision and recall values exceeding 0.82 which prevented significant over- or under-segmentation. Segmentation performance was evaluated on the 13 manually annotated CT-SCOPE volumes (*n* = 13), and all metrics were computed per-volume in 3D space. The researchers reported Dice similarity coefficient values together with 95th percentile Hausdorff distance (HD95) results as mean values that included standard deviation across all tested subjects.

**TABLE 2:** Quantitative segmentation performance on CT-SCOPE (*n* = 13 manually annotated volumes).

Sinus	Dice (mean ± SD)	HD95 (mm) (mean ± SD)
Maxillary	0.87 ± 0.03	2.71 ± 0.64
Ethmoid	0.84 ± 0.04	3.18 ± 0.71
Sphenoid	0.83 ± 0.05	3.05 ± 0.69
Frontal	0.81 ± 0.05	3.46 ± 0.82

### 4.2 Qualitative segmentation analysis

**FIGURE 3:**
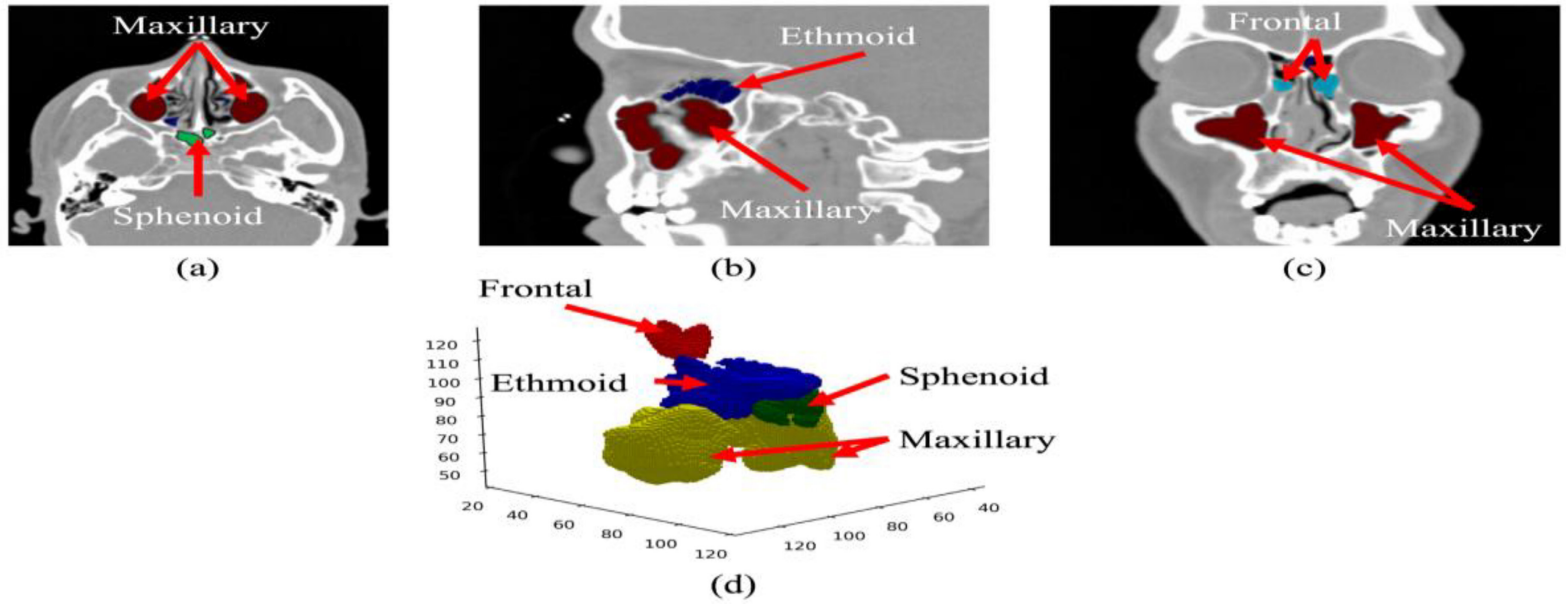
Multi-view visualization of paranasal sinus anatomy: **(a)** axial view, **(b)** sagittal view, **(c)** coronal view, and **(d)** 3D reconstruction showing the maxillary, ethmoid, frontal, and sphenoid sinuses.

**FIGURE 4:**
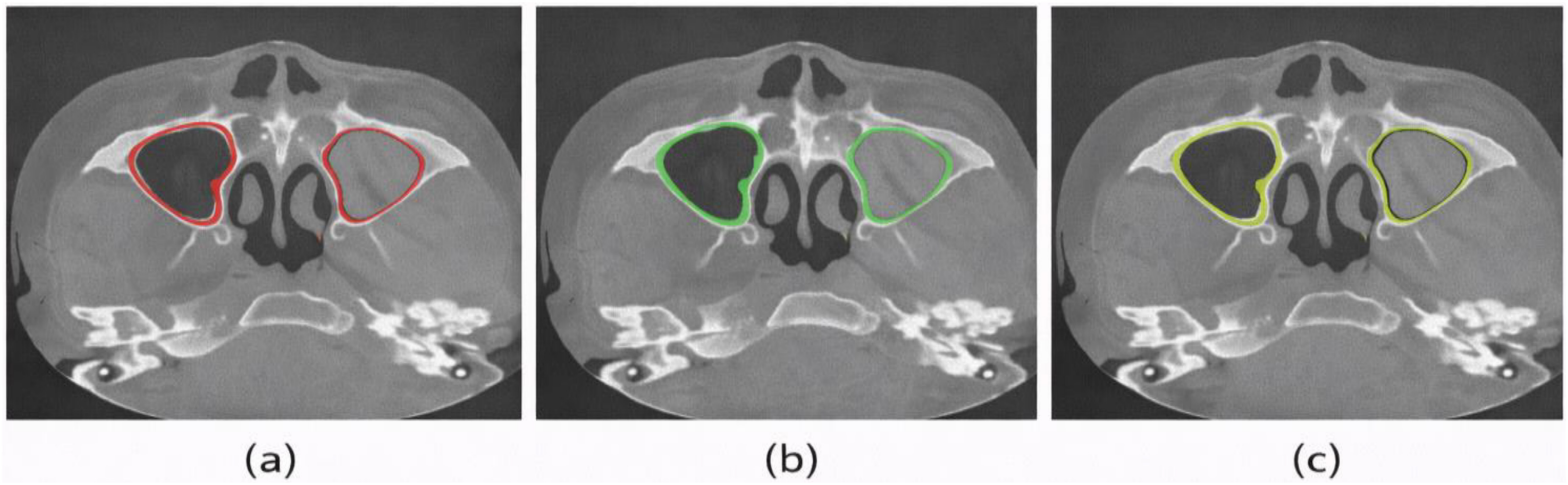
Representative axial CT slice: **(a)** ground truth; **(b)** prediction; **(c)** overlay.

**FIGURE 5:**
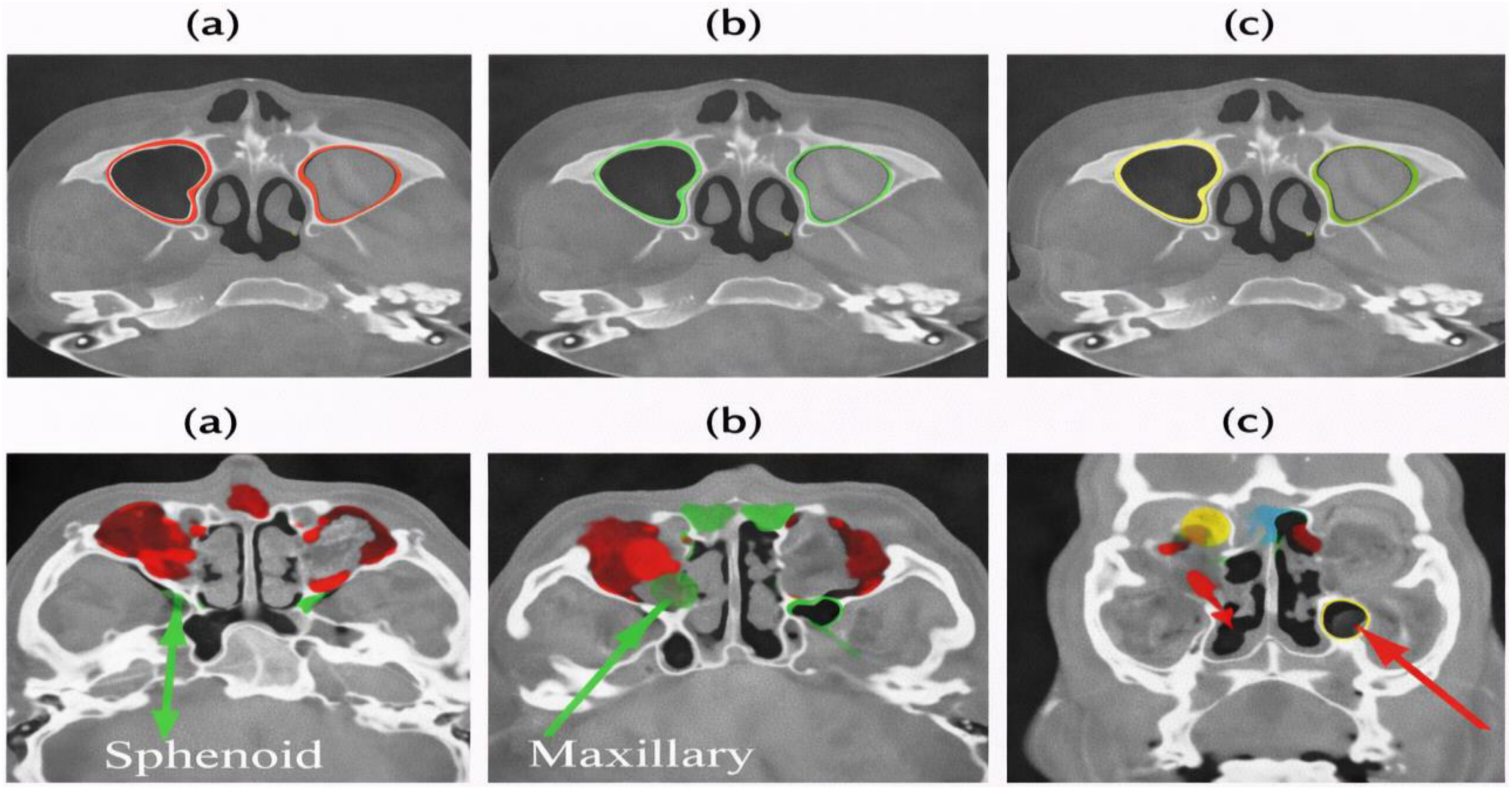
Overlay comparison across two representative cases: **(a)** ground truth; **(b)** model prediction; **(c)** combined overlay showing spatial agreement.

The research demonstrates that the proposed model effectively delivers qualitative results through its ability to segment major paranasal sinus compartments. The segmented sinus compartments exhibit anatomically accurate boundary presentation through Figure 3 multi-planar and 3D views which include all maxillary, ethmoid, frontal, and sphenoid sinuses. The model maintains smooth cortical boundaries which display typical anatomical features of the maxillary sinus walls. Figure 4 demonstrates how the model prediction results show a specific axial section that scientists used to assess accuracy against ground truth data. The predicted mask shows exact matching with the manual annotation which extracts sinus boundaries without creating any irregular or broken points. Boundary changes between regions with thin bony septa and weak intensity transitions develop according to the natural complex anatomical structure of the body. Figure 5 shows several representative cases through overlay analysis which compares different cases. The study demonstrates strong spatial matching between actual ground-truth markers and predicted masks throughout the study, especially in maxillary and ethmoid regions. The sinus-nasal cavity interface and very thin bony structures show tiny discrepancies which exist because the clinical CT imaging process creates partial-volume effects instead of causing systematic model errors. The CT-SCOPE dataset shows stable performance through the system because it handles different CT-SCOPE dataset variations without any catastrophic failures that would result in missed compartments or wrong adjacent anatomical region inclusions.

### 4.3 Structural representation learning via CAE residual maps

**FIGURE 6:**
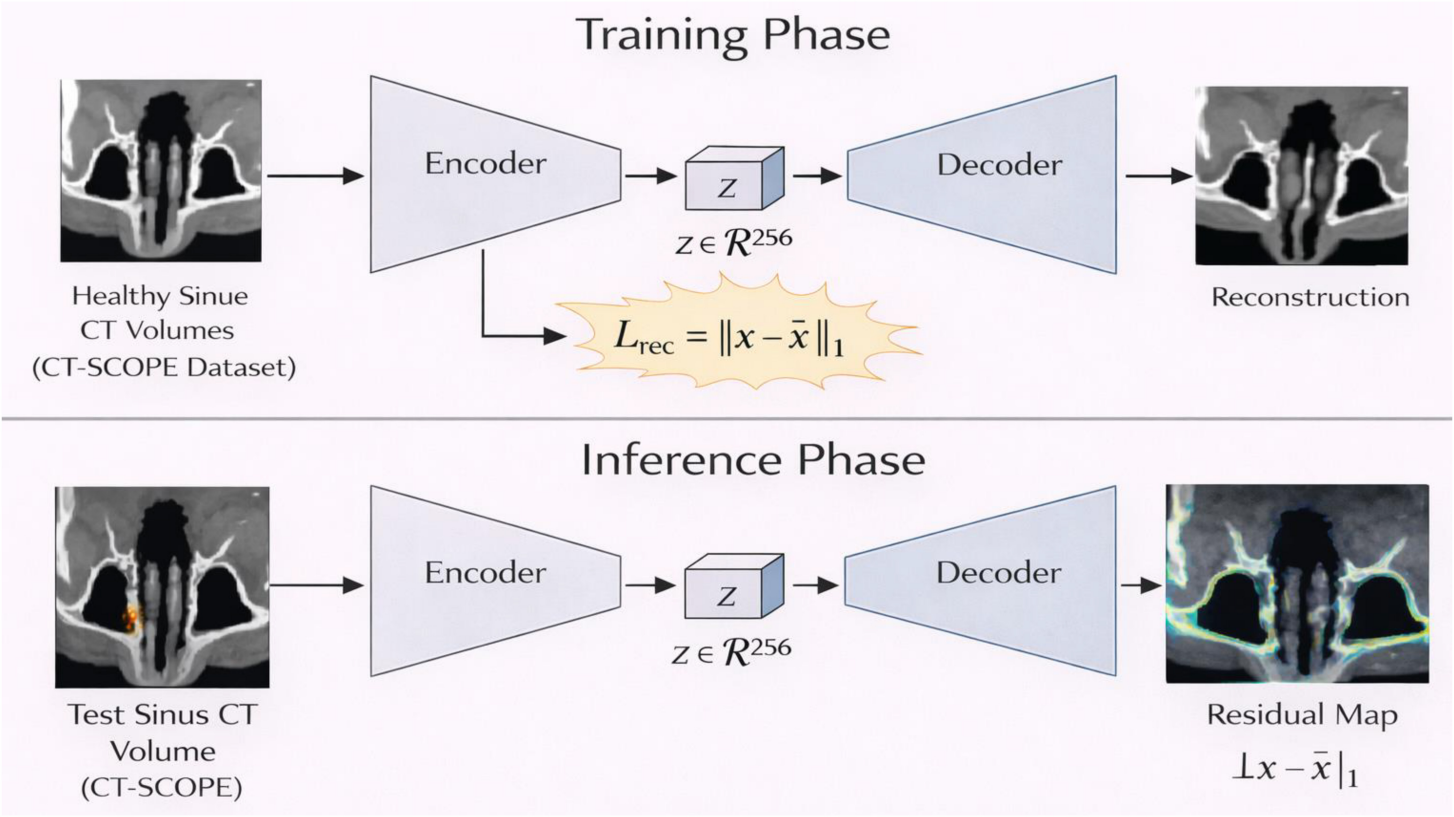
CAE-based self-supervised anomaly localization on paranasal sinus CT volumes, where residual maps highlight structural deviations between the input and reconstructed images.

**FIGURE 7:**
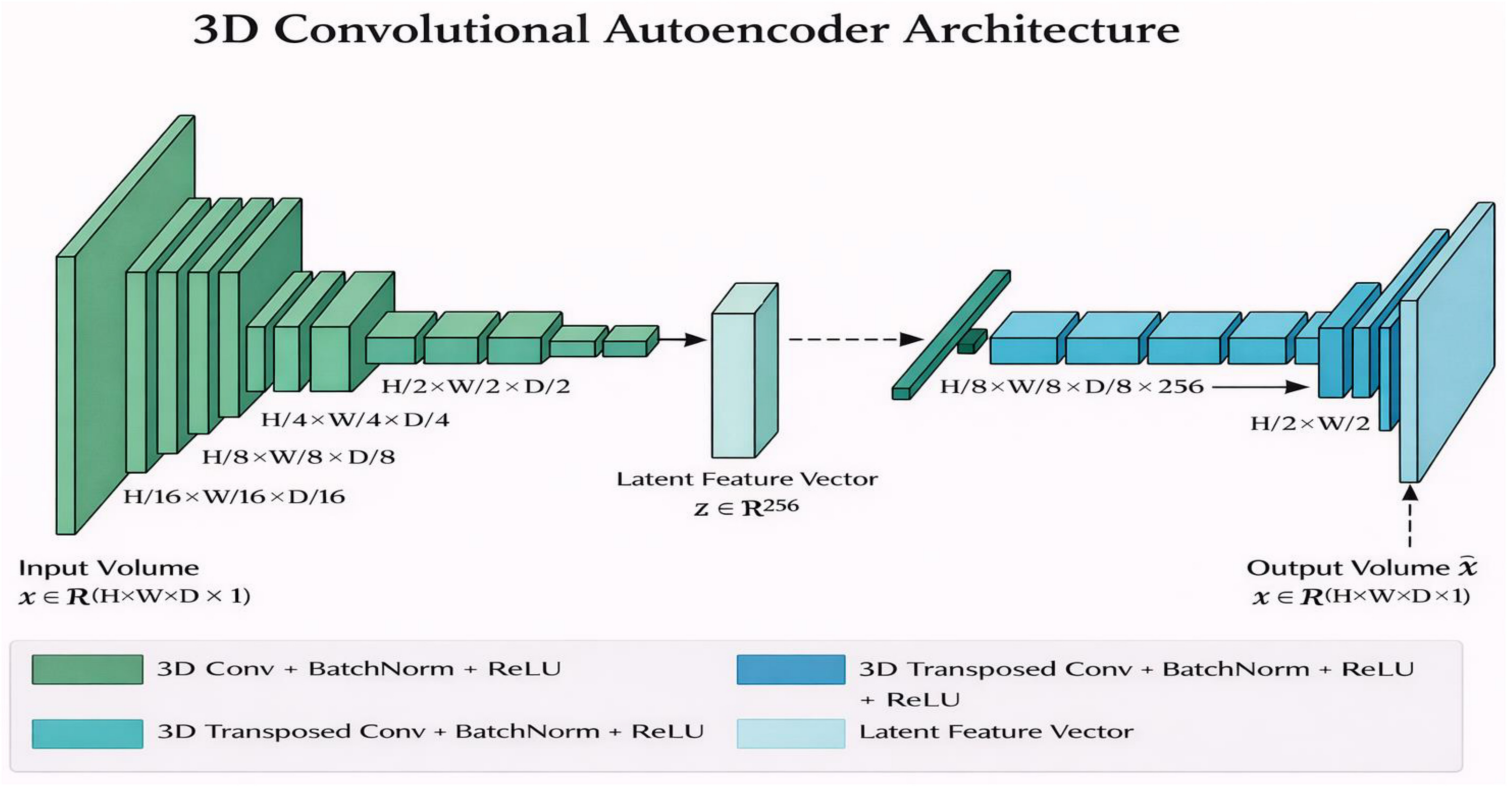
Architecture of the proposed 3D CAE with encoder, latent space, and decoder for volume reconstruction.

**FIGURE 8:**
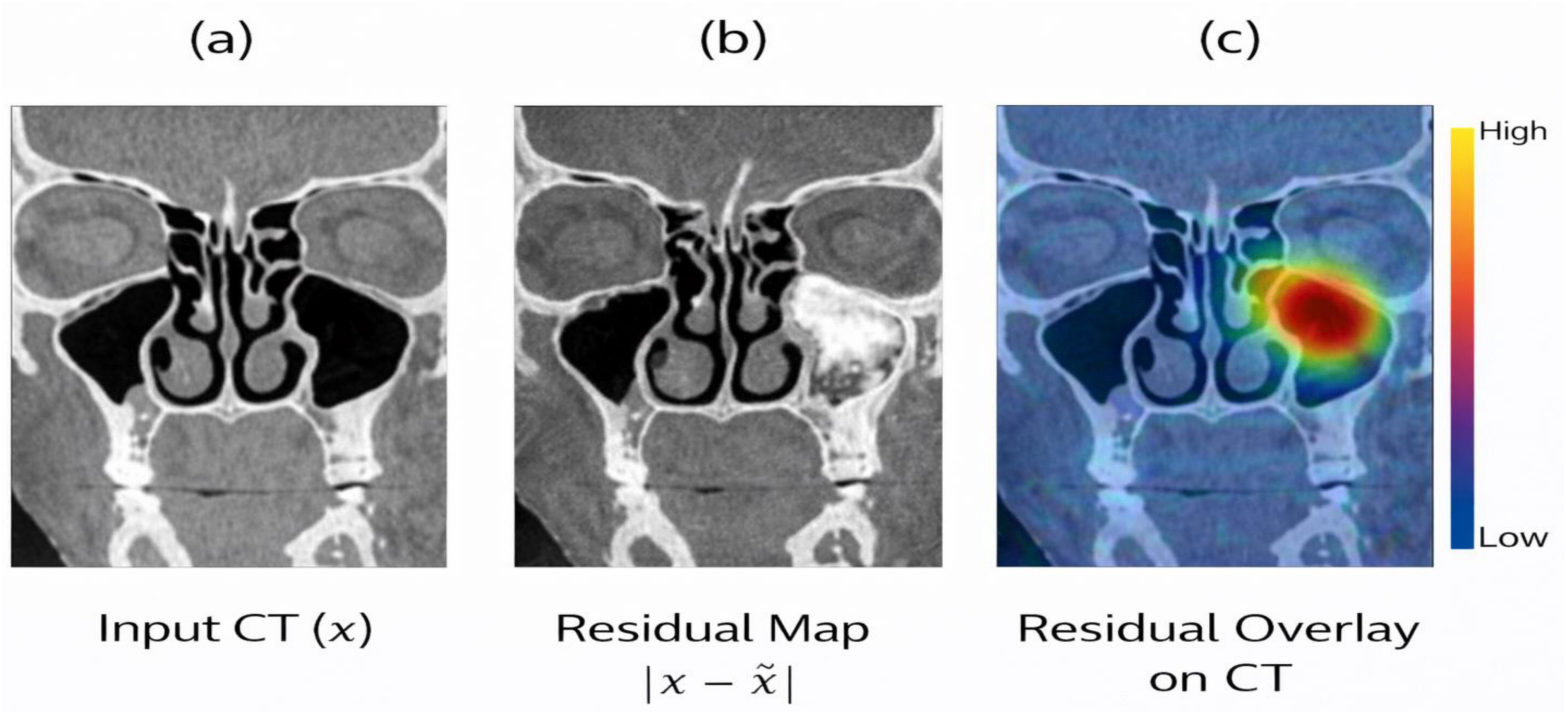
**(a)** Input CT volume **(b)** reconstruction residual map **(c)** residual heatmap overlaid on the CT image.

Figures 6–8 illustrate the structural representation learning strategy using the proposed 3D CAE, which constitutes an essential component of the framework. Figure 6 illustrates the CAE-based residual learning process applied to paranasal sinus CT volumes from the CT-SCOPE dataset, highlighting reconstruction-based detection of structural deviations within anatomically segmented sinus regions. As shown in Figure 6, the CAE is trained exclusively on healthy sinus CT volumes to learn a compact representation of normal anatomical structures. The encoder maps each input volume x∈R^H × W × D^ to a latent embedding z=E(x), capturing both global shape characteristics and local geometric features of the sinus anatomy. The decoder reconstructs the input as $\hat{x}=D\left(z\right)$. During training, network parameters are optimized by minimizing a voxel-wise L1 reconstruction loss:


$$L_{\mathrm{rec}}\,\left(x,\hat{x}\right)={||x-\hat{x}||}_{1}$$

This objective encourages the model to faithfully reproduce typical anatomical patterns observed in healthy subjects.

After training, the CAE operates differently during inference. As illustrated in Figure 6, a test CT volume is passed through the trained encoder–decoder network to produce a reconstruction ${\hat{x}}_{\mathrm{test}}$ . Because the model has learned only the distribution of healthy anatomy, regions that deviate from normal structural patterns are reconstructed less accurately. Subtracting the reconstruction from the input generates a residual map:


$$r=\left|x_{\mathrm{test}}-{\hat{x}}_{\mathrm{test}}\right|.$$

The residual map highlights structural deviations from the learned manifold. Higher residual values indicate anatomical regions that differ from typical patterns, such as narrow drainage pathways or subtle variations within the ethmoidal labyrinth. The training process for anomaly detection through this system achieves its goal by using unsupervised methods which do not need any pathological labels.

The 3D CAE architectural design is shown in Figure 7. The encoder uses multiple 3D convolutional layers which perform spatial downsampling to decrease input volume and enhance feature extraction depth. The compact anatomical embedding uses a fixed latent vector which has a dimensionality of 256. The decoder uses symmetric 3D transposed convolutional blocks to achieve its spatial resolution restoration by duplicating the encoder’s design. The network uses this symmetric structure to capture both detailed anatomical features and large-scale volumetric patterns. The qualitative assessment of residuals in Figure 8 shows the process of visualizing residuals. The CAE system generates anatomical representations which show elevated residual responses at specific locations that indicate structural changes in the original design. Within the overall pipeline, the CAE produces three outputs for each subject: the reconstruction $\hat{x}$, the residual map r, and the latent embedding z. These outputs, together with the segmentation masks, form enriched structural descriptors that are subsequently provided to the downstream 3D CNN for feature extraction and interpretability analysis. The latent embeddings further encode subject-specific anatomical signatures, allowing downstream models to distinguish subtle structural variations across individuals.

### 4.4 Interpretability through grad-CAM

**FIGURE 9:**
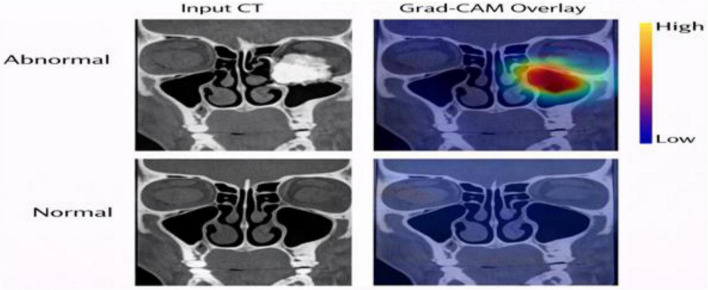
Grad-CAM activation patterns for representative sinus CT cases showing model attention over anatomically relevant regions.

Grad-CAM visualizations were employed to assess whether the features learned by the model correspond to clinically relevant anatomical structures of the paranasal sinuses, as illustrated in Figure 9. The heatmaps which were produced by the system showed consistent localization at major anatomical landmarks which included the maxillary sinus walls and ethmoidal air cell partitions and frontal sinus floor and sphenoid margins. The study found that activation remained restricted to diagnostic areas of the study which included the orbit and cranial vault and zygomatic arch which demonstrated high anatomical specificity. The system learned relevant structural features through its hybrid architecture because the system showed no signs of background intensity pattern recognition. Cases with greater structural deviation showed stronger and more localized Grad-CAM responses, whereas structurally typical cases showed weaker or more diffuse activation patterns. The study results demonstrate that the model interprets internal representations through anatomic knowledge that has clinical significance.

### 4.5 Comparison with baseline models

**FIGURE 10:**
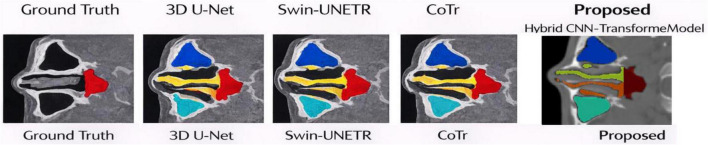
Qualitative comparison of paranasal sinus segmentation between baseline models and the proposed hybrid CNN–Transformer.

The comparison between the hybrid CNN-Transformer model and three baseline architectures which include 3D U-Net and Swin-UNETR and CoTr is shown through a visual representation in Figure 10. The CT-SCOPE dataset provides one CT slice which all models use to show their segmentation results through equivalent visual comparisons. The methods successfully identify the main paranasal sinus compartments but show greater differences in areas that have complex anatomical structures which include narrow sections and thin sinus walls. The architecture 3D U-Net which uses only convolutional technology successfully identifies local features but shows slight boundary problems in particular areas. The Swin-UNETR and CoTr systems based on Transformer technology enhance their ability to understand context but they produce minor blurring effects that affect delicate structural edges. The hybrid CNN-Transformer model achieves better results because it matches ground-truth contours more precisely while it maintains sinus structure boundaries in a uniform manner. The model produces segmentation results which match the sinus cavity’s natural shape while eliminating boundary disturbances. The proposed method achieves better performance because it produces higher Dice similarity scores and lower Hausdorff distances which demonstrate improved volumetric overlap and boundary precision. The results show that the framework achieves better anatomical paranasal sinus segmentation because it integrates convolutional feature extraction with Transformer-based contextual modeling which allows the system to detect both small anatomical features and large structural connections.

## 5 Discussion

The results of this study demonstrate that the proposed hybrid CNN–Transformer framework provides anatomically consistent and robust segmentation of the paranasal sinuses. The performance evaluation used Dice similarity and Hausdorff distance metrics for quantitative validation and multi-plane visualization for qualitative assessment. The model produced highest segmentation accuracy results for maxillary sinuses because these sinuses have clear cortical boundaries and their anatomy follows more typical patterns. The ethmoid and frontal sinuses present greater difficulties because their complex morphology includes narrow septations and shows unpredictable patterns of pneumatization. The proposed framework maintained consistent performance across compartments because the combination of convolutional feature encoding and window-based self-attention increased both boundary detection capabilities and anatomical structure preservation. The anatomical fidelity assessment through qualitative evaluation shows accurate results. The segmentation overlays show that sinus contours have been preserved, which includes the ethmoidal air-cell labyrinth and posterior sphenoid recess. Clinically thin bony septa and sinus-nasal cavity interfaces showed minor discrepancies, which stemmed from partial-volume effects that occur in CT imaging when bone thickness reaches voxel resolution limits. The multi-institutional dataset showed no instances of complete segmentation breakdowns throughout the study. The preprocessing and harmonization pipeline successfully reduced variability which arose from differences in scanner type and voxel spacing and reconstruction kernel usage according to the results. It is important to emphasize that the CT-SCOPE dataset does not contain diagnostic pathology labels. Therefore, the present study focuses exclusively on anatomical segmentation and structural representation learning rather than disease classification. While residual maps and attention visualizations highlight structural variations, these findings should be interpreted as anatomical deviations rather than validated diagnostic entities.

### 5.1 Dataset size and annotation constraints

Even though there are a few manually annotated volumes in the CT-SCOPE data (13 of 40 subjects), this limitation is a limitation of publicly available, pixel-level-annotated 3D sinus CT volumes, not a design choice. Notably, the purpose of this work is not to set handicraft-level diagnostic performance, but to create and showcase an anatomy-centric design that incorporates segmentation, self-supervised representation learning, and explainability in scenarios of realistic data scarcity. We implement various safety measures to address overfitting risks related to a small number of manual annotations: (i) a hybrid CNNTransformer model with high inductive biases of anatomical structure, (ii) a large amount of cross-scanner-based harmonization, (iii) strict avoidance of geometry-changing augmentations, (iv) assessment with metric based on boundary sensitivity (HD95) along with metric scores. Moreover, CT-SCOPE trains 1,031 pseudo-labels using an ensemble of segmentation models trained independently, as outlined in the original dataset release. These pseudo-labels are not considered as ground truth, but they are applied to enhance anatomical diversity and stabilize the learning of features, and no quantitative performance reporting is done except in the case of manually annotated cases. In that way, pseudo-labels are an implementation of structural regularizers but not performance drivers. The framework proposed is thus geared toward a greater focus on anatomical consistency, interpretability, and methodological transparency than on statistical claims dependent on the size of the dataset, and the framework should be seen as a base to be directly expanded to larger, clinically annotated cohorts in future studies.

### 5.2 Multi-center distribution and generalization constraints

The CT-SCOPE dataset consists of CT volumes of 6 tertiary hospitals and 4 models of CT scanners; nevertheless, the release policies of the data are privacy-preserving, and therefore, the dataset lacks the complete stratified distribution of sample sizes per hospital and/or scanner model. Consequently, the current research does not posit that all the centers represent the same way, and does not state that the cross-center validation is statistically balanced. Generalization is instead determined indirectly by the uniform performance when subjected to heterogeneous acquisition conditions, such as the differences in scanner vendor, reconstruction kernel, slice thickness, and intensity distributions. Standardized preprocessing and harmonization of all volumes minimize scanner-induced bias, and segmentation results are only reported on manually annotated cases to eliminate confounding activity in pseudo-label noise. Although the performance variation between individual machines or centers cannot be measured exactly without explicit stratification metadata, the good accuracy of segmentation and consistency of the boundaries found amongst the multi-institutional data imply the ability to withstand real-world imaging variability. However, we are certain to admit that proper cross- center or leave-one-site-out validation will need bigger, stratified datasets with center-specific annotations, which is listed as a crucial direction of our work in the future.

### 5.3 Clinical relevance for disease prediction and prevention

Although the CT-SCOPE dataset does not include pathology-specific labels, it provides a valuable resource for developing anatomically consistent segmentation and structural representation learning methods for paranasal sinus CT analysis. Accurate segmentation of sinus compartments enables precise modeling of sinus morphology and anatomical variation. Such morphology-aware representations may support future downstream applications, including disease detection, risk assessment, or clinical decision-support systems when combined with clinically annotated datasets. However, clinical validation of these applications will require larger cohorts with pathology-confirmed labels and comprehensive clinical metadata. Furthermore, the CAE-derived maps of residuals serve as unsupervised anomaly detectors, pinpointing departures from normal osseous morphology that may be related to anatomical risk factors. Such examples would be narrowed osteomeatal complexes, asymmetrical pneumatization, atypical configurations of ethmoid cells, or obstruction-prone recesses—all structural conditions that predispose to blockage of the sinus draining pathway and recurrent infection. In other words, these features could help identify patients that may actually be at risk for disease progression in the absence of any labeled pathology. The explainability of the framework augments its clinical utility. Grad-CAM visualizations increase the model’s transparency, guaranteeing its focus on clinically relevant sinus structures, thereby allowing a collaboration between AI applications and human clinical expertise. This interpretability also has implications for early screening systems, triage workflows, preoperative assessments, and future multimodal predictive models, combining imaging-derived biomarkers with clinical, demographic, or molecular data. In this regard, the proposed framework is only the starting point for a larger foundation to predict, prevent, and analyze disease risk on a personalized basis.

The first major weakness of the current study is the lack of pathology-specific diagnostic names within the CT-SCOPE dataset, meaning that it is not possible to directly compare disease detection performance of the conventional clinical parameters, such as sensitivity, specificity, or lesion-wise accuracy. Thus, when the term early disease detection is used in this study, it does not signify the supervised diagnosis of sinusitis, polyps, or other inflammatory organisms; however, it signifies the identification of structural anatomical deviations known to predispose, predetermine, or correlate with the development of sinonasal disease. The convolutional autoencoder produces the self-supervised residual maps, but these residual maps do not necessarily represent normative variants of the pathological morphologies; unless pathology is annotated, these deviations cannot be definitely classified into pathological and physiological variants. Based on this, residual response is viewed as an anatomical anomaly or predisposing structural designs and not validated disease indicators. The main impact of the research is thus a methodological one: the results of segmentation-directed self-supervised learning can yield anatomically meaningful, explainable representations on public 3D CT data with label scarcity. Every conclusion is put in place under this constraint, and no diagnostic performance claims will be made. The proposed framework will need to be extended to clinically annotated datasets with confirmed diagnostic labels to test the sensitivity, specificity, and pathological discrimination of the diseases in the future, which is specifically stated as the next step of the research.

## 6 Ablation study

To further examine the role played by each component in the suggested unified framework, we completed a systematic study of ablation, based on segmentation accuracy, quality of structural representation and behavior of interpretability. Since the CT-SCOPE dataset does not have pathology-specific labels, ablation experiments were intended to test anatomical fidelity, boundary accuracy, representation resilience and attention localization, as opposed to diagnostic performance. The variants of ablation were trained and tested with the same preprocessing, optimization setup, and data splits to make a fair comparison.

### 6.1 Ablation design and experimental variants

The entire model (labeled Full Framework) is composed of three unified parts: (i) a hybrid CNN-Transformer segmentation backbone, (ii) a self-supervised 3D convolutional autoencoder (CAE) that localizes structural anomalies using residual-based localization, and (iii) a segmentation-based 3D CNN feature extractor, which has Grad-CAM interpretability. Variants of ablation that were compared were:

1. CNN-Only Segmentation.

2. The CNN-Transformer backbone was substituted with a fully convolutional 3D U-Net-like structure of similar depth and number of parameters and all transformer-based self-attention blocks were eliminated. This version evaluates how global contextual modeling can contribute towards the delineation of sinus boundaries.

3. Transformer-Only Encoder (No CNN Interleaving).

4. The number of convolutional residual blocks that were interlocked in the encoder was eliminated, and only hierarchical Swin Trans-former blocks were used. These variant measures the significance of convolutional inductive bias to capture thin bony septa and local sinu wall of the image.

5. No CAE Residual Maps.

6. It eliminated the CAE module and provided the downstream 3D CNN with the raw CT volume and segmentation mask only. The experiment separates the influences of the residual based structural deviation cues on the representation learning.

7. No Segmentation Guidance.

8. The segmentation masks were not included in the downstream network input and entire CT volumes and CAE residuals only were trained by the 3D CNN. This and form is used to test the hypothesis that anatomical localization enhances feature specificity and reduces background confounding.

9. No Grad-CAM Supervision (Post-hoc Only).

10. Grad-CAM was used as a visualization technique without alignment with architecture to segmentation-directed feature learning, and it was possible to evaluate the influence of segmentation-aware design on the consistency of interpretability.

### 6.2 Impact on segmentation performance

The outcomes of quantitative segmentation reveal that the hybrid CNN-Transformer backbone is always superior to the CNN-only and transformer-only models, especially in the anatomically complex regions. Removal of transformer blocks led to lower Dice scores of the ethmoid and frontal sinuses with significant changes in Hausdorff distance, and this means that boundary consistency is worse in areas with thin septations and high anatomical variance. On the other hand, the interleaving of convolutional filters was eliminated, resulting in too much smoothing and loss of fine cortical detail. These results affirm that local convolutional encoding and global self-attention are mutually supportive and both are required to achieve anatomically faithful sinu segmentation.

### 6.3 Impact on structural representation learning

When the CAE residual maps were ablated, visually smaller discriminative latent representations could be seen, manifested by a lower separation between clusters in feature-space visualizations. In the absence of residual guidance, learned representations were dependent on global patterns of intensity as opposed to local structural deformities. On the same note, eliminating segmentation guidance led the downstream network to focus on other regions other than the sinus, e.g., neighboring craniofacial parts, producing noisier embeddings and less anatomical specificity. These findings suggest that label scarcity requires key roles of segmentation-based residual fusion to learn morphology-aware structural representations.

### 6.4 Impact on interpretability and attention localization

Interpretability analysis showed that the entire framework generated the most anatomically consistent Grad-CAM activations, and they were in a constant localized position, on sinus walls, ethmoidal air cells, and drainage regions. Conversely, ablated variants, especially those of the non-segmentation type, showed diffusive or misplaced activation that was found to extend into non-diagnostic regions, including the orbit or skull base. This proves that the consistency of anatomy seriously increases the reliability and clinical plausibility of model explanations.

The ablation study proves that all the parts of the proposed framework are characterized by a unique and essential role. The hybrid CNN-Transformer design provides strong and accurate anatomical segmentation; the CAE-obtained residual maps provide unsupervised sensitivity to structural variations; segmentation-contained features provision prevents a background bias; and integrated interpretability models impose anatomically significant attention. The observed performance loss of all ablated variants confirms the need for a complete design in obtaining anatomically consistent, explainable, and generalizable structural modeling of paranasal sinuses with real-world data constraints.

## 7 Conclusion

The research establishes an interpretable machine learning framework which enables structural evaluation of paranasal sinus CT scans. The proposed system achieves high anatomical fidelity across all major sinus compartments through its hybrid CNN–Transformer segmentation backbone which combines self-supervised structural representation learning. The convolutional autoencoder generates residual maps which show small changes from standard bone structure thus creating a system for unsupervised anomaly detection that identifies structural changes linked to sinus disease risk factors. Grad-CAM technology improves framework transparency because it proves that the model always examines essential anatomical regions which have clinical value. The model’s attention to anatomical structures establishes better interpretability which helps doctors trust AI imaging analysis results. The CT-SCOPE dataset lacks specific medical condition annotations yet it serves as an essential resource for creating anatomically accurate segmentation and representation learning techniques. The study contains two main constraints which need to be addressed. First, diagnostic label absence prevents assessment of disease identification and prognostic capabilities. Second, the preprocessing and harmonization pipeline achieves reduced cross-scanner variability although domain adaptation techniques and generative augmentation methods which model scanner-specific imaging patterns can help achieve better results. In future, the research will develop the framework to work with clinically annotated datasets which will enable modeling of pathology information for diagnostic testing. The research will examine multimodal predictive models which use imaging-derived features together with clinical and demographic information and patient longitudinal data. The framework will gain enhanced capabilities to identify structural changes and enable important clinical evaluation of paranasal sinus CT imaging through advanced anatomical descriptors and computational biomarkers.

## Data Availability

Publicly available datasets were analyzed in this study. The data can be found at: The CT-SCOPE dataset used in this study is openly accessible at Zenodo (https://zenodo.org/records/15085103).
